# Multidimensional performance enablers of Ethiopian community-based health extension program: A scoping review

**DOI:** 10.1371/journal.pone.0324377

**Published:** 2025-06-05

**Authors:** Kora Tushune Godana, Zewdie Birhanu, Gugsa Nemera, Kasahun Eba, Fikadu Balcha, Yibeltal Assefa, Wim V. Damme, Peter Decat

**Affiliations:** 1 Department of Health Policy and Management, Public Health Faculty, Jimma University Jimma, Jimma, Ethiopia; 2 Department of Health, Behavior and Society, Faculty of Public Health, Jimma University, Jimma, Ethiopia; 3 Department of Nursing, Faculty of Health Sciences, Jimma University, Jimma, Ethiopia; 4 Department of Environmental Health Sciences and Technology, Faculty of Public Health, Jimma University, Jimma, Ethiopia; 5 School of Public Health, The University of Queensland, Queensland, Australia; 6 Department of Public Health, Institute of Tropical Medicine, Antwerp, Belgium; 7 Department of Public Health and Primary Care, Faculty of Medicine and Health Sciences, University of Gent, Ghent, Belgium; JSI, ETHIOPIA

## Abstract

**Introduction:**

Ethiopian Health Extension Program (HEP) aims to improve access to primary care, yet suboptimal health outcomes persist, indicating the need to optimize HEP implementation. This scoping review aims is to identify and synthesize evidence on the enabling factors that influence the Ethiopian HEP and Health Extension Workers (HEWs) performance.

**Methods:**

A comprehensive search strategy was employed across multiple databases, including PubMed, EMBASE, Web of Science, and institutional websites. The review included studies published since January 2003, encompassing quantitative, qualitative, and mixed-methods researchs. The PRISMA flow diagram guideline was utilized for reporting of a review process and JBI methodology guideline was used for data extraction. Data extraction was conducted using Atlas.ti software, capturing verbatim excerpts and applying thematic coding to illustrate key findings.

**Results:**

This review identified key multidimensional enablers of the Ethiopian HEP that collectively enhance the program’s performance. Motivational factors for HEWs play a critical role, encompassing intrinsic desires and extrinsic factors including opportunities for career growth and fair compensation. Additionally, the review identified the importance of continuous capacity building to enhance the skills of HEWs and program performance. The review also highlights the positive impact of diversifying HEP services including customizing the HEP services to the varied contexts and integrating male HEWs to enhance program reach and acceptance. Engaging communities through organized campaigns, stakeholder coordination, and leveraging local structures enable and strengthen the program’s effectiveness. Finally, the availability of essential resources is crucial for HEWs to provide timely and appropriate care. These multidimensional enabling factors form pathways for HEWs and HEP to create supportive environment and capability that significantly enhances HEP performance.

**Conclusions:**

Intrinsic and extrinsic motivations, community engagement, clinical nurse inclusion, male HEWs participation, mentorship and improving living conditions of HEWs enhances the HEWs’ effectiveness and program performance.

## Introduction

The Health Extension Program (HEP) in Ethiopia has made a significant impact by increasing essential healthcare services, resulting in better maternal and child health outcomes, and increased community health awareness. However, the program faces challenges including inadequate salaries and incentives; lack of training, support and supervision; limited opportunities for career development; high workloads; resource constraint; and mismatch in community expectation and skills of Health Extension Workers (HEWs). To address these challenges, the HEP optimization roadmap outlines strategic shifts, including improvements in human resources, infrastructure upgrades, and the transformation of remote health posts into comprehensive facilities. It also advocates for merging nearby health posts with catchment health centers or hospitals. Importantly, the roadmap stresses the need for community engagement, proposing initiatives like the Village Health Leaders structure to ensure local participation in the healthcare system [1].

Health Extension Workers as community health workers are increasingly being recognized as an essential cadre for the achievement of Sustainable Development Goals (SDGs), Universal Health Coverage (UHC), and the future health agenda, especially in settings where resources are constrained and human resources are limited. To this end, community-based health programs are platform and vehicle for delivering essential health services such as maternal and child health services to the communities [2–4]. If managed and implemented well, community-based strategies improve health outcomes, including increasing antenatal care (ANC) visits, micronutrient supplement uptakes, improving birth planning, and increasing care-seeking for pregnancy complications and institutional delivery [3].

Based on the guiding principle of Primary Health Care (PHC), Ethiopia launched a community-based healthcare system in 2003, the HEP to increase access to primary care and improve population health outcomes by reaching the grassroots community [5,6]. The HEP assigns two female salaried HEWs to implement eighteen packages of health services organized under four main themes: 1) Disease Prevention and Control, 2) Health Education and Communication, 3) Hygiene and Environmental Sanitation, and 4) Family Health through widely dispersed community health posts, home visits, and other means of outreach strategies [5–7]. The HEP is based on the assumption that access to and quality of essential preventive and selected curative primary health care for grassroots communities can be improved through the transfer of health knowledge and skills to communities and households using those trained, salaried, female cadres, as major workforce. Most importantly, the HEP aims to realize UHC through which all Ethiopians can access needed health services, including health promotion, disease prevention, treatment of minor conditions, rehabilitation and palliative care. Over time, the program has gone through several phases and evolutions with the intention of meeting the changing needs of communities and respond to ever-changing health challenges. For instance, in principle, the HEWs who were initially trained for one year has been upgraded to level IV training, and the health service packages they are supposed to deliver has also been upgraded to include basic curative services and other essential packages such as chronic diseases, and mental health aspects [8,9]. Moreover, in 2010, the HEP introduced a new community engagement strategy, the women and men-centered Health Development Army (HDA), to promote participatory community engagement and adoption of healthy lifestyles and to improve the uptake of critical health services. The HDA is composed of five members and referred to as one-to-five women networks [10]. Health Extension Workers and local administrators, in collaboration with community members, identify and train model households who become leader of the one-to-five network. All members of HDA are expected to participate in the implementation of HEP, particularly support HEWs by identifying and linking pregnant women to the health system [11]. In 2020, Ethiopia developed its HEP optimization roadmap 2020–2035 in alignment with the Health Sector Development and Investment Plan (HSDIP). The roadmap contains several transformative initiatives that will be rolled out in phases towards the goal of achieving UHC [12].

Although significant progress has been documented, several poor health outcomes are still high in Ethiopia, suggesting the uptake of the HEP packages is still not optimal despite decades of implementation and efforts. The UN estimates the maternal mortality ratio (MMR) in Ethiopia for 2020 at 267 maternal deaths per 100,000 live births, showing significant progress in reducing maternal mortality, though still far from the SDG target of 70. According to the Mini Demographic and Health survey report, in Ethiopia, 74% of women aged 15–49 had a live birth in the 5 years before the survey received ANC from a skilled provider for their most recent birth. However, the fact that only 43% of women had at least four ANC visits during their last pregnancy highlights the urgent need for improvement in maternal healthcare [13].

Over the past decade, a substantial amount of research has been conducted on HEP, yet there is still need to understand factors that could enable the performance of the HEP cadres and the program. Given that HEP is the backbone of the national health system and will remain the primary strategy to enhance Ethiopian health status, it is imperative to map and synthesize existing evidence to construct the enablers of HEWs/HEP over the decades of implementations experience. In this regard, it is essential to understand how the multiple enabling factors with its multiple levels operate to influence the provisions of HEP services positively. However, in-depth evidence is lacking on the subject, and an evidence base need to be built up to through examine literatures to map what factors could enable the performance of the program, which lead to building up the pathways to better performance optimization of the performance of the HEP/HEWs. Such knowledge will contribute to identifying critical intervention pathways for optimization of the performance in Ethiopia and similar settings. Therefore, it is timely and imperative to identify and document important enabling factors for the HEP in Ethiopia. Understanding critical enabling factors to HEP/HWs would have several implications and significance to the program

This scoping review maps evidence on enabling factors that influence the implementation of the HEP in Ethiopia, which could offer insights that support its effective execution. It also contributes to the existing literature and informs strategies to strengthen HEP implementation in Ethiopia and similar community-based health programs elsewhere.

## Methods

This scoping review was conducted using the methodology developed by the Joanna Briggs Institute 98 (JBI) [14]. The review followed a priori protocol, which is publicly available in Open Science Framework (https://osf.io/z2wf6/metadata/osf).

**Deviations from the Protocol:** There were some deviations were made from the original protocol. Firstly, the authors expanded the scope of the review question to comprehensively explore the multidimensional performance enablers of Ethiopian HEP. This broader approach aimed to construct and leverage available literature documenting enablers on this program since its inception. In contrast to the original protocol, which had a narrower focus, the current review prioritizes identifying key enabling factors that are essential for enhancing and optimizing HEP implementation. The authors believe that this expansion to include questions about performance enablers allows for a deeper understanding of the multifaceted elements that empower and facilitate the program’s success.

Our analysis in this review focused specifically on identifying the enabling and facilitating factors that influence the performance of HEWs and the HEP. The review process involved developing specific inclusion criteria based on the Population, Concept, and Context (PCC) framework [15,16]. Population, Concept, and Context allowed us to identify the target populations involved in or impacted by the HEP, specify the key concepts related to the performance of HEWs and the HEP, and define the contexts and settings relevant to the review. The following sections detail the specifications used to conduct the review, with a focus on mapping the enabling and facilitating factors of the Ethiopian HEP.

### Participants

This review focused on studies and reports related to the factors that enable and facilitate the performance of HEWs and the HEP in Ethiopia. It included findings related to the review objectives from a broad range of study populations, including health extension workers, community members and groups, program implementers, partners, stakeholders, health workers, policymakers, and program managers.

### Concept

The primary concept of interest in this scoping review was to assess the extent of literature available on the enablers related to the Ethiopian HEP and HEWs. These enablers are potentially multidimensional encompassing individual, organizational, social, economic and political aspects. Specifically, factors such as inputs and resources, infrastructure and facilities, financial factors, leadership and governance, support and communication, and the work conditions and environment of HEWs were considered. We identified and reported themes and factors that serve as enablers or facilitators, helping HEWs perform their jobs and duties more effectively.

### Context

This scoping review focused on studies and reports on the Ethiopian HEP, covering all administrative regions and city administrations in Ethiopia.

### Types of Sources

This scoping review considered a wide range of sources, including quantitative, qualitative, and mixed-methods studies, as well as published and grey literature. The quantitative studies included all types of experimental and quasi-experimental designs, such as randomized controlled trials, non-randomized controlled trials, before-and-after studies, and interrupted time-series studies. Additionally, analytical observational studies, including prospective and retrospective cohort studies, case-control studies, and analytical cross-sectional studies, were considered for inclusion. Descriptive observational study designs, such as case series, individual case reports, and descriptive cross-sectional studies were also included.

Qualitative studies focusing on qualitative data were considered, encompassing designs such as phenomenology, grounded theory, ethnography, qualitative description, action research, and feminist research. Furthermore, the review included reports with valuable lessons, discussion articles, and review articles, such as systematic reviews on the HEP and HEWs, particularly those addressing enablers and facilitators that enhance program performance.

### Search strategy

The search strategy was designed to locate both published and unpublished studies related to the studies on Ethiopian HEP. A three-step approach was employed for this review. First, an initial, limited search was conducted in MEDLINE (PubMed) and EMBASE to identify relevant articles. The text words in the titles and abstracts of these articles, along with the index terms used to describe them, were analyzed to develop a comprehensive search strategy for PubMed (see S1 File). This search strategy, including all identified keywords and index terms, was then tailored for each database and information source included in the review. Additionally, the reference lists of all included sources of evidence were screened to identify further relevant studies. The review included studies published in English from January 2003 to January 2024.

The databases searched were PubMed, EMBASE, Web of Science, Cochrane Database of Systematic Reviews, JBI Evidence Synthesis, and African Journals Online (AJOL). To identify unpublished studies and gray literature, searches were conducted in ProQuest Dissertations and Theses, Google Scholar, and institutional websites such as Jimma University, Addis Ababa University, the Ethiopian Public Health Institute, and the Ministry of Health.

### Study/Source of evidence selection

After completing the search, all identified citations were compiled and uploaded into EndNote version 20 (Clarivate Analytics, PA, USA), where duplicates were removed. Following a pilot test, the titles and abstracts were screened by five independent reviewers to assess their relevance against the inclusion criteria for the review. Sources deemed potentially relevant were retrieved in full, and their citation details were imported into Atlas.ti version 7.5.18 (GmbH, Berlin, Germany) to extract study characteristics and key findings. The full text of selected citations was thoroughly evaluated against the inclusion criteria by five independent reviewers. Reasons for excluding sources that did not meet the inclusion criteria at the full-text stage were documented and reported in the scoping review. Any disagreements between reviewers during each stage of the selection process were resolved through discussion or with the involvement of an additional reviewer. The results of the search and the study inclusion process were fully detailed in the final scoping review and presented in a Preferred Reporting Items for Systematic Reviews and Meta-Analyses extension for scoping reviews (PRISMA-ScR) flow diagram [17].

### Data extraction

Data from the papers included in the scoping review were extracted by four independent reviewers (KT, ZB, KE, GN, FB) following the JBI guidance for conducting scoping reviews. The extracted data included specific details about study characteristics, methods, participants, concepts, context, and key findings relevant to the review questions. The extraction process was supported by Atlas.ti software, which was used to capture segments of the papers as quotations, providing verbatim excerpts that illustrate themes, concepts, or findings within the data. Additionally, we used codes—words or short phrases that symbolically assign essence-capturing meaning—to reference the selected quotations.

The draft data extraction tool was modified and refined as needed throughout the process of extracting data from each included source of evidence. Any disagreements between reviewers were resolved through discussion or with the involvement of an additional reviewer. When necessary, the authors of papers were contacted to request missing or additional data.

### Data analysis and presentation

The results of the scoping review are organized and presented using thematic and sub-theme analysis, supported by narrative descriptions of the findings extracted from the included papers. The thematic areas are aligned with the objectives of the scoping review, highlighting and characterizing the multidimensional enabling factors that contribute to the improved performance of the HEP. Finally, these multidimensional themes are conceptualized and mapped in a diagram that illustrates a pathway to enhanced performance.

## Results

### Study selection

**Fig 1:**
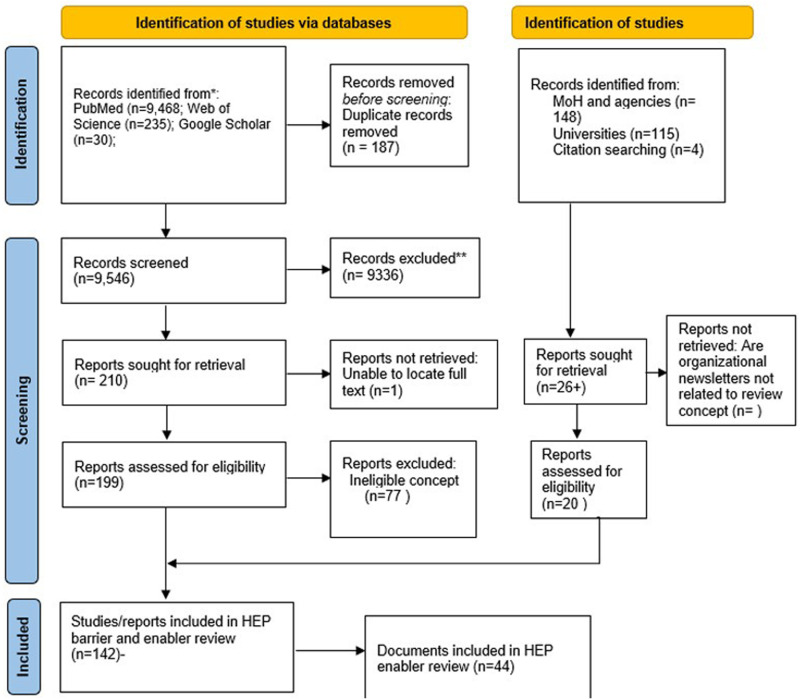
PRSIMA flow diagram of study selection process.

A total of 9733 records were identified through database searches and an additional 149 records were found via other methods after the removal of 187 duplicates, 9694 records were screened based on title and abstract, and 9336 of those were excluded. The full texts of 210 records were assessed for eligibility, and 11 were excluded. Reasons for exclusion were due to context, concept, population and content irrelevances. A total of 141 documents included in barriers and enablers review out of which 44 documents were reviewed and included in this review. The search results and study selection process are summarized in Fig 1.

### Characteristics of included documents

The scoping review included 44 documents related to enablers of HEP performances in Ethiopia, comprising a mix of 25 published articles [18–42], 14 grey literatures [43–56] and others [57–61]. Various study designs were represented, with 10 observational studies [21,25,29,34–36,38,57,58,61], two experimental studies [30,43], 10 qualitative studies [18,19,21,23,27,28,31,32,37,60], 10 policy document analyses [44,45,47–49,51–54,62], 5 reviews [20,33,39–41], 5 mixed [24,26,42,46,59] and two documents did not reported their study design [50,56]. The studies were conducted across diverse settings: 15 studies were conducted in community-based settings [20–22,24,27–30,34–36,43,57,61], four in facility-based settings [18,25,38,60], three at system-level [47,48,52] and eight conducted in mixed settings [31,32,37,63–67], for nine documents it was not applicable [39,40,44,48,49,53–56] while five documents did not report the study settings [19,35,42,50,58].

Regarding the study context, 16 studies focused on rural areas [18,21,22,25,27,29,31,35–38,43,44,57,60,61], four on urban settings [23,24,35,59], eight covered both rural and urban settings [20,26,28,32,45,46,48,52], while 12 were not applicable [33,39–41,47–49,51,53–56] and 4 did not specified [19,44,50,58]. The region’s most frequently represented in the review were Oromia [6,21,28,29,57,59] studies involved multiple regions [8,18,20,22,24,32,43,44] and nationally-focused studies [18,26,33,39,40,46–55,68,69]. Other regions such as Amhara (4 studies) [25,27,34,35,60,70] Tigray (2 studies) [31,38] and Sidama (3 studies) [19,36,37] were less frequently represented in the included documents. Additionally, three studies focused on other smaller or less commonly represented regions, including Benishangul-Gumuz, Addis Ababa, and the Southern Nations, Nationalities, and Peoples’ Region (SNNPR) [23,30,61]. Some studies did not specify a particular region [56,57]. This suggests that while some areas were well-represented, others had relatively limited coverage in the included documents. The populations studied included HEWs and healthcare providers (HCPs) in seven studies [18,25,26,31,38,58,59], child caretakers in three studies [21,34,35] a mixed population of various stakeholders [19,20,22–24,27–30,32,36,37,43,46,52,57,60,61] and other document-based populations [9,33,39,41,45,48,49,53], while 8 studies did not specify the population [44,47,48,50,51,54–56].

## Review findings

### Enablers of health extension program

The review identified six central themes and related sub-themes interconnected and collectively contribute to the overall performance of the Ethiopian HEP. The success of the HEP relies heavily on various enabling factors, including the motivation and job satisfaction of HEWs, capacity building, community empowerment and engagement, stakeholder engagement, and trust building for fostering effective communication, collaboration, and participation among community members, healthcare providers, and other stakeholder; and availability of necessary resources and infrastructures. All of these elements are crucial in ensuring the successful implementation and impact of the HEP. The following sub-sections provide a detailed description of themes and sub-themes.

### Motivation factors: Intrinsic and extrinsic motivators as driver of performance

The review identified intrinsic and extrinsic motivational factors as crucial drivers of HEWs performance and job satisfaction and thereby ensuring the successful implementation of HEP. According to the review, HEWs are driven by a deep-seated desire and passion to serve their communities, experience a sense of fulfillment of their societal duty and obligations through their work [21,32,40,59], and drives positive behavioral shifts that align with the goals and objectives of HEP. Moreover, this intrinsic motivation is a key factor in transcending HEWs’ interactions, fostering a supportive environment for the program, increasing the utilization of healthcare facilities, raising service utilization rates, and transforming community health attitudes [32], ultimately enhancing the performance of HEP [20,53,59].

Furthermore, the review reveals that several extrinsic factors play a significant role in enhancing the performance of HEWs. These factors include opportunities for career advancement, fair compensation for their work, the presence of incentive mechanisms, and recognition from the community [42,71]. Notably, providing career development opportunities for HEWs, which enable them to progress from entry-level positions (level 3) to higher levels (level 4) [21,40,51,71], helps to upgrade their knowledge and skills, motivating them to excel in their roles and ultimately improving the performance of HEPs [20,71].Multiple sources also reported that better payment, incentives and satisfaction with their job are linked to better performance of HEWs and the program [20,33,42,71,72].

### Diversification, contextualization and standardization of HEP

This review highlights the importance of diversifying the Ethiopian HEP to better address the diverse health needs of different population segments. Specifically, studies suggest that expanding the scope of health services provided at health posts to include curative services would enhance extrinsic enabling factors, such as community engagement, and service acceptance, while aligning with intrinsic factors like HEWs desire to meet community expectation and improve HEP performance. However, this addition should be approached cautiously, as some HEWs may worry that this could weaken the program’s focus on promotive and preventive services [43,44]. The review emphasizes the importance of contextualizing the HEP to the diverse geographical, cultural, and socioeconomic landscapes such as urban, agrarian, and pastoralist areas which fosters intrinsic motivation through culturally sensitive delivery and reinforcing extrinsic support through justifiable resource allocation [49].

The other need for diversification of HEP is to include male in the HEP workforce which addresses extrinsic logistic barrier like transportation challenges in remote areas. This strategy also strengthens intrinsic community trust in conservative settings, where male workers may enhance cultural acceptability. Some reviewed articles indicated that engaging male enhances the program’s reach and performance because males were perceived to withstand challenges related outreach activities such as hard to reach arras and transporting logistics at absence of transportation services [43,44]. The review also indicated that the integration or assignment of clinical nurses into health posts level could enhance extrinsic enabling factors such as technical capacity of HEWs and boost the HEP performances. The review highlighted the need to standardize the roles, numbers, and types of volunteer community health workers, including Community Health Agents (CHAs) and Trained/Traditional Birth Attendants (TBAs), to ensure consistent and HEP service delivery across different settings [44].

### Ongoing capacity building and support system

The review revealed that HEWs who are adequately capacitated and well-supported are better equipped to deliver effective health services to the community, which in turn enhances the performance of the HEP and ultimately leading to improved health outcomes [19,25,43,58,59,73,74]. For instance, up skilling and updating HEWs with best practices such as improved access to various sources of information, reference materials, guidelines, and updates on health developments enhances HEWs capacity to perform HEP activities [25,42,43,47,48,50]. In addition, number of studies revealed ongoing on job capacity building interventions and support system like regular on-the-job training and refresher courses either comprehensive [39] or specifics like on how to manage vaccine cold chain [75], ANC, clean and safe delivery, immunization, family planning could help them to excel in their roles, leading to maximize HEP performance [43,58].

Effective leadership including frequent interactions and healthy relationship with supervisors who provide constructive feedback (encouraging, written, oral, and problem-solving) and regular performance reviews and feedback [20,41,76] create a learning and supportive environment that could enable HEWs to perform well their duties [58]. For example, one article showed that open, clear, and consistent communication between HEWs and their supervisors fosters mutual understanding and trust, better opportunity for regular updates, feedback, and discussions about challenges and successes contribute to a supportive work environment [20,55]. Additionally, the reviewed articles showed that supportive supervision and guidance are better achieved in collaboration with stakeholders that contribute additional resources and expertise [19,59]. Supervisors who provide adequate support, training, and resources help HEWs perform their duties effectively [25,43,47].

### Effectively engaging communities and community structures

In this review several sources showed that effectively engaging communities, community structures, and local stakeholders is crucial in enhancing the performance of HEWs in Ethiopia. Organized community-based campaign helps to build a positive and supportive environment for better performance of HEWs [77,78]. Moreover, such campaigns play a crucial role in disseminating information regarding available health services, thereby encouraging community members to utilize HEP services more effectively [78]. Furthermore, the review findings indicate that proactive community involvement in the planning phase of the HEP cultivates a sense of ownership, thereby enhancing the propensity for community members to utilize HEP services, support HEWs, and adopt recommended health practices [77]. For example, one of the reviewed articles reported that early engagement of the community played a critical role in ensuring regular antenatal care visits and facility-based deliveries [18].

### Well-coordinated stakeholder engagement and participation enhance HEWs performance

The review vividly revealed that involving and collaborating with various stakeholders such as Agricultural Development Agents (DA), Nongovernmental Organization (NGOs), and schools play a crucial role in supporting HEP [24,28,36,40,41,43,49,59,61,77]. Fully engaged Stakeholders support HEP through various means, including social support, resource mobilization, and motivation of community improve access to healthcare services and promote healthcare-seeking practices [18,24,43,57]. Several articles reported that effectively coordinated collaborative effort between HEWs and various stakeholders enhances HEWs/HEP performance [7,43,59,77,79]. However, it is also important to acknowledge that if not well coordinated such collaboration increase the workload of HEWs and there by negatively affect HEP performance [3,24,77].

### Leveraging community structures, networks, and leadership enhance HEW/HEP performance *Community structures and networks as engagement platforms*

The review indicated that utilizing local existing grassroots community structures, networks and leadership can improve HEWs performance and impact. The diverse community structures situated at community level include the women groups— women volunteer groups or Women’s Development Army (WDA) also called HDAs, “Afosha” (community traditional self-help system), men groups, local leaders, village/kebele leaders and cabinets and other community organization system [41,45,57]. These structure serve as platform and means of sharing and exchanging health information between communities and the HEWs; building partnerships between HEWs and communities; and actively work to connect the HEWs with the community’s needs and foster collaboration for better HEP outcomes [45,57]. Additionally, these structures served as agent to create opportunities for interaction and knowledge exchange between HEWs and community leaders, ultimately strengthening the uptake of HEP services [28]. These multi-pronged community structures, influential figures, and community networks supported and maximized HEP performance [23]. For instance, an article reported that the WDA serves as a critical platform and a vital resource within the community for engaging women in promoting positive health behaviors and facilitating knowledge sharing [49]. In this regard, HDAs networks acted as crucial information in linking pregnant women to health facilities [18,22,48,49] by facilitating referrals [18] and providing prenatal care guidance [23]. Additionally, HDAs raise community awareness about health and HEP [22].

***Leadership roles in mobilizing resources and driving engagement*** According to the review, Kebele leaders and local governing bodies play a crucial role in linking HEWs with households, to facilitate access to healthcare and mobilize resources. They promote community engagement in HEP activities, and reinforce social mobilization committees for health activities [18,49,57]. For instance, one of the reviewed articles suggested that the close collaboration between HEWs, kebele councils, and HDAs boosts the performance of HEP by streamlining referrals and resource allocation [30].

#### Model families as behavioral change catalyst.

The review also revealed that trained and certified model families serve as a change agent for behavioral change by advocating healthcare service in the community, raising awareness, creating social influences, promoting healthy practices, sharing best practices and lesson learned and enhance positive health behavior, ultimately contributing to the success of HEP ([34,35,37,59,77].

#### Inclusion of influential community figures.

The review also emphasized that involving significant others figures like mothers-in-law, men, traditional birth attendants, and religious leaders significantly increase healthcare service utilization of institutional delivery, ANC, and PNC utilization [18,31].

### Trusting relationship between community and HEWs performance

The review highlighted the importance of several key qualities the HEWs should embrace in order to establish trust with the communities they serve. These qualities include effective communication skills, organizational skills, interpersonal collaboration abilities, strong work ethic, regular availability of HEWs on their duty workplace, relevant work experience and competency which could foster a positive relationship with the community, ultimately contributing to the success of HEP [19,22,24].

### Availability of resources, supporting facilities and basic infrastructures

This review highlights the crucial role of adequate resources, including medical supplies (e.g., essential medicines, contraceptives, vaccines, and consumables) and basic yet essential equipment (e.g., thermometers, fetoscopes, and stethoscopes), in enabling HEWs to effectively perform their duties [18,19,46,49,58,59]. For instance, one of the documents reviewed reported that health posts which got drug supply are 7.19 times functional than when compared to those who did not [58]. The review also indicated that the availability of office materials (such as furniture, stationaries), electric supply, water supply, and telephone at health post as well as transportation for HEWs, maternity waiting homes, and exempted medical services at nearby health centers could enhanced HEWs performance [3,18,19,26,43,46].

**Fig 2:**
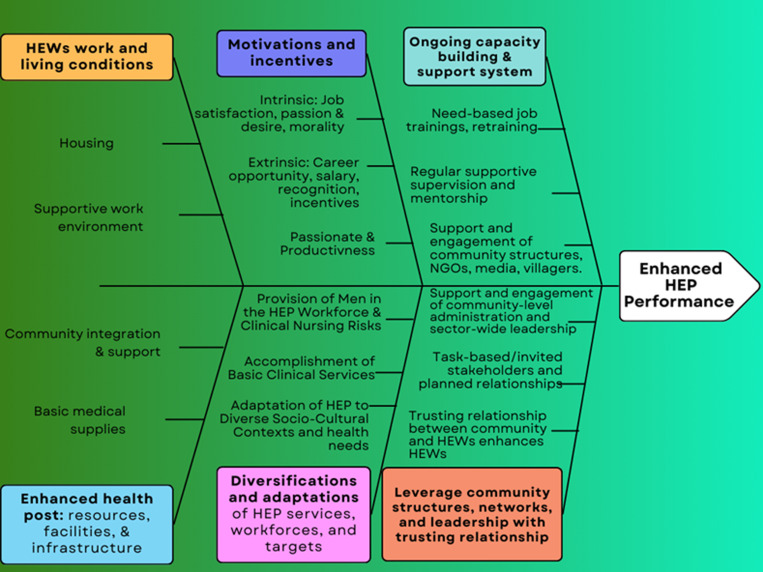
Conceptual Framework of Enabling Factors Influencing Health Extension Workers’ Performance. The figure highlights the conceptual framework’s focus on factors that enhance the performance of HEWs and the HEP as emerged through synthesis of evidence from the review. As depicted in the figure there are multidimensional enabling and facilitating factors contributing to pathways for enhanced or better HEWs/HEP performances.

Improved living and supportive work environments for HEWs have been shown to positively impact their retention, performance, and the overall effectiveness of HEP [39,40,80]. For instance, one of the reviewed documents found that providing better housing for HEWs led to a 15% increase in the number of households visited and 20% improvement in the quality of health services delivered. Similarly, improved working conditions and support lower attrition by 25% when compared to their peers with poorer work environments [76]. The factors that enhance the performance of HEWs and the HEP is highlighted in the conceptual framework in Fig 2.

## Discussion

This scoping review synthesized the enabling factors that enhance the performance of HEWs and Ethiopian HEP. The findings were conceptualized into eight major themes and sub-themes: motivation factors, diversification and adaptation of services, capacity building and support, community engagement, trusting relationships, and resource availability. Intrinsic and extrinsic motivation are crucial drivers of HEW performance. Diversifying and adapting HEP services, workforces, and target populations improve program reach and impact. Continuous capacity building and robust support systems are essential for maintaining HEW performance. Engaging communities, utilizing local structures and leadership, and coordinating stakeholders are key strategies for improving HEP outcomes. Trusting relationships between the community and HEWs fosters collaboration and program success. Additionally, improved resources, facilities, and infrastructure at health posts, along with better living and working conditions for HEWs, enhance their performance and the program’s efficiency. These themes highlight strategies to optimize HEW performance and improve the effectiveness of the Health Extension Program.

The review indicated that Intrinsic and extrinsic motivational factors play a substantial role in enabling HEWs to perform their jobs effectively, thereby contributing to the enhanced performance of the HEP. Consequently, from the review, several intrinsically driven motivation mechanisms that motivate HEWs to engage in HEP for their own sake rather than for external rewards, such as being satisfied with their job, inherent passion and desire to serve the society they came from and were born from (community and social connections) conceived as fulfilling societal obligations and sense of belongingness were documented as powerful motivators of HEWs to perform their jobs. When these workers experience job satisfaction, they are more likely to be engaged, committed, and effective in their roles, leading to improved retention rates and better program outcomes. Many HEWs are driven by a strong desire to help their communities and make a positive impact, which can lead to increased effort and dedication. Additionally, fulfilling societal obligations is a powerful motivator, as these workers often view their roles as a way to give back to their communities and uphold cultural values. These factors could lead to higher job satisfaction and motivation, such as recognition, opportunities for advancement, and the nature of the work itself [81]. These intrinsic factors, on the other hand, could play an important role in enabling and boosting Hew’s commitment and performance. It is essential to focus on their intrinsic motivations by ensuring and reinforcing their sense of societal obligation and belonging and their work is meaningful, offering growth opportunities, and supporting their community ties and social connections, integrating cultural and personal values to ensure that the HEP work aligns with the health HEWs’ personal values and cultural beliefs, reinforcing their sense of purpose.

The review highlighted that extrinsic motivation factors can be powerful drivers of Hew’s performance. Factors such as career development opportunities, incentives (both in-kind and financial), competitive compensation, and recognition from supervisors, peers, and the broader community were identified as particularly effective motivators. Providing access to training, education, and pathways to advancement can effectively increase motivation and job performance through improved career development opportunities. By catering to HEWs’ desires for professional growth, financial rewards, and social recognition, organizations can tap into powerful extrinsic motivators that complement intrinsic sources of motivation. A multifaceted approach that addresses extrinsic and intrinsic factors is likely to yield the best results in driving sustained high performance. It should be noted that combining intrinsic and extrinsic motivational factors is key to unlocking the full potential of this vital cadre of frontline health providers to enable them to perform better performance. By addressing intrinsic and extrinsic motivational factors, the Ethiopia HEP can create a holistic approach to supporting and empowering HEWs, ultimately leading to improved healthcare service delivery and better health outcomes for their communities.

Studies in low-and middle-income settings and other contexts indicated that a combination of intrinsic and extrinsic motivational elements is essential for enabling and maintaining high-quality performance among community health workers in resource-constrained settings. A systematic review found that intrinsic motivators such as altruism and community embeddedness and extrinsic factors like financial incentives and career advancement opportunities were critical for driving strong performance [39]. Similarly, a qualitative study in Tanzania by Greenspan et al. identified key intrinsic motivators for community health workers, including a sense of purpose, personal growth, and connection to their communities; extrinsic factors like financial remuneration, supplies, and pathways for career development were also important motivators [81]. They emphasized the need for a comprehensive approach to improving community health worker performance, addressing intrinsic motivators (e.g., recognition) and extrinsic incentives (e.g., compensation, training). This multi-pronged strategy is crucial for optimizing the contributions of community health workers and sustaining their engagement over the long term [82].

The review revealed several interesting findings regarding how diversifying the Ethiopian HEP could enable the HEWs and the overall program to perform more effectively. The **concept of diversification** was explored from multiple perspectives: **Contextualization and Adaptation of** the HEP to the varied socio-cultural contexts, health needs, and experiences of different communities - whether urban, rural, pastoral, or agrarian - was considered crucial. Accommodating this diversity in settings and community characteristics would make the program more responsive to local realities. This suggests that the HEP should have the flexibility to tailor service delivery approaches, training, and support structures based on the unique circumstances of different regions, urban/rural areas, and pastoral/agrarian communities. It should be noted that adapting the HEP to local contexts would require decentralized planning and decision-making to empower regional and district-level implementers, and this will have an important policy implication because it calls for programmatic, practical, and policy-level adaptations to enable the HEP to fulfill its potential through having a flexible nature, and enabling framework that allows for contextual adaptation of the HEP. This may require revisiting national guidelines and standards to provide the necessary latitude for local decision-making regarding HEP, such as creating policies that should also support the diversification of the HEWs cadre, the formal engagement of community volunteers, and the strategic integration of traditional birth attendants within the realm of HEP-community ecosystem.

Diversifying the HEP workforce was also highlighted as an essential factor. This included incorporating male HEP cadres and service providers and integrating clinical nurses to strengthen the program and empower Hew’s technical capacity. Making the HEP more gender-equitable by including males as HEP cadres was also cited as a crucial enabler for the HEWs and the overall program performance. The inclusions of males as an enabling factor for the HEWs and the program was viewed in terms of the males’ quality, such as the ability to work in challenging work conditions, such as travelling long distances by carrying medical necessary medical supplies such as vaccines; ability travel at night to serve the community at home such as in case of emergency and delivery assistance; and also able to influence and mobilize community when broader community engagement is needed. Evidence from other settings also suggests that having a gender-balanced mix of community health workers (CHWs) can positively impact their performance and effectiveness: can improve access and acceptability of services, especially for addressing sensitive health issues such as gender-matching between CHWs and clients as a factor that positively influenced CHW performance [39]; can better connect with and gain the trust of diverse community members [81]; enables to reach marginalized groups more effectively [83]; enable complementary skills and perspectives and better opportunity to provide more holistic and comprehensive services care [84].

On the other hand, proper integration of community volunteers, especially the Women Development Armies and traditional birth attendants with clearly defined roles and engagement guidelines, and establishing formal engagement mechanisms were identified as a crucial enabling factor and would help leverage this additional resource for optimization of the HEP. Engaging these community-based resources could help leverage their unique insights and connections to influence the community. Particularly, the review suggests that engaging traditional birth attendants (TBAs), whose trusted relationships with communities could complement the formal HEP, is also warranted and could significantly help HEWs perform better. This proper integration, engagement, and leveraging of their knowledge should be carefully managed to ensure that TBAs complement rather than compete with formal health services. This involves integrating TBAs into the health system through training and collaboration. Policies should formalize TBA involvement, ensuring their roles are well-defined and contribute positively to maternal and child health without undermining the quality of care. Studies in different similar connect reflected that integrating traditional birth attendants (TBAs) into community health worker (CHW) programs can enhance CHW performance by leveraging TBAs’ community trust and connections, positively influence CHW effectiveness improve CHWs’ motivation and job satisfaction [39,81,82]

Another crucial enabling factor the review identified was the inclusion of curative services within the HEP’s mandate, which could enhance HEWs and the HEP acceptance as it meets community expectations and contributes to their performance. The evidence from elsewhere on whether the inclusion of curative or clinical services into community health programs that primarily focus on preventive and promotive services has a positive or negative impact on program performance is mixed. A systematic review [39] found that including curative services was one of the factors that positively influenced community health worker (CHW) performance. This is because it can improve community acceptance and utilization of the CHW program. A study in Pakistan showed that integrating [85] curative services for childhood illnesses into the Lady Health Worker program enhanced the program’s effectiveness in reducing child mortality. However, there are potential risks in integrating curative components into the program because it could potentially undermine and neglect the preventive and promotive focus of the CHW program and can divert their attention from core public health functions, potentially weakening the overall impact of the program [39,86,87]. The integration of curative services should be approached cautiously to maintain the program’s preventive and promotive focus and addressed through policy guidance. The key is to find the right balance and ensure that the expansion of services does not undermine the core strengths and focus of the community health program. Careful program design, training, supervision, and monitoring are crucial to maximize the benefits while mitigating the potential risks.

Altogether, this concept of diversification of HEP as enabling factors requires creating regionalized HEP packages and training of an HEP workforce varied by the regional context, moving away from a one-size-fits-all strategy towards **a “tailored”** or **“customized”** strategy that responds to the specific requirements, characteristics, or conditions of different individuals or groups.

The evidence from the review highlights that ongoing, regular and continuous capacity building and support are crucial for enabling HEWs to perform their duties effectively. Continuous on-the-job training and refresher courses are vital as they address emerging needs, fill knowledge gaps, and reinforce key competencies to keep HEWs up-to-date. Supportive supervision and mentorship also play essential roles. Consistent, constructive feedback from supervisors through regular performance reviews, clinical mentorship, and guidance helps HEWs tackle challenges and improve practices. Positive reinforcement and encouragement further foster a learning environment. Open communication and trust-building are equally important. Frequent, transparent communication between HEWs and their supervisors allows for discussing challenges, sharing successes, receiving timely feedback, and cultivating a relationship of mutual understanding and trust. Collaborative, multi-stakeholder supportive supervision was cited as a key enabling factor. This joint supervision could help HEWs to gain adequate support, advice, mentors, necessary resources and expertise during the supervision session in a well-coordinated and consistent manner, which is, in contrast, when stakeholders separately in an uncoordinated manner try to support and approach the HEWs. Thus, the review reflected that coordinating efforts in supervision and support systems, together with ensuring HEWs have access to necessary information, reference materials, and updates as part of supportive supervision, are important enablers. The evidence from the literature strongly supports the notion that ongoing and needs-based capacity-building strategies such as continuous and tailored training, constructive feedback and supportive supervision [39,81,84], collaborative support combined with adequate resourcing is can create a supportive work environment that empowers CHWs to effectively carry out their responsibilities and deliver quality community health services. This notion of continuous capacity building and support systems as critical enablers would have several key implications for Ministry of Health stakeholders supporting the Ethiopian Health Extension Program (HEP). Firstly, the Ministry should ensure regular, tailored training and refresher courses for HEWs to keep them equipped to handle evolving community health challenges. Secondly, a robust supportive supervision system, with constructive feedback, mentorship, and positive reinforcement, is crucial for helping HEWs improve their practices and navigate challenges. Investing in HEW supervisor capacity is key. Thirdly, facilitating transparent communication between HEWs and supervisors will build mutual trust and understanding, allowing HEWs to discuss issues and receive timely feedback. The Ministry should also engage diverse stakeholders to coordinate efforts and mobilize additional support for HEWs.

This review demonstrates that community structure, networks, and leadership are vital enablers for HEWs and the success of Ethiopia’s HEP. These elements provide a strong foundation for HEP operations and significantly enhance the program’s effectiveness when communities actively plan and execute HEP activities. Key components of this involvement include the engagement of model families and the support of local leadership. Active participation by community networks, notably the Women’s Development Army (WDA) and various community groups, is essential. These groups offer ongoing support and serve as a bridge between HEWs/HEP and the community, facilitating information exchange and community mobilization. Households trained and graduated as model families play a crucial role by serving as role models and encouraging their communities to adopt healthy practices. Formal leadership at the village/kebele and sub-village levels is a significant enabler for HEWs. To maximize the impact of the HEP, it is crucial to leverage community networks and structures, along with committed and motivated community-level leaders, both formal and informal. The HEP should focus on providing clear directions, guidance, and support to engage community actors and structures for improved outcomes effectively. From its inception, the HEP has integrated organized community engagement with the women-centered HDA strategy, serving as a strong enabling structure for HEWs. This presents an opportunity to capitalize on these community networks to boost HEWs and the program further. By integrating community structures, networks, and leadership, HEWs can enhance their capacity to deliver comprehensive and culturally sensitive health services. This approach helps build stronger partnerships, address contextual barriers, and ensure that the HEP is responsive to the needs and priorities of the communities they serve, ultimately enhancing the performance and impact of HEWs and the HEP. By leveraging these community-level resources, community health workers can better tailor their interventions, access necessary support, and build community trust, ultimately enhancing their capacity to deliver effective health services. Evidence from various LMICs highlights the critical role of community-level factors in enabling the work of community health workers. In Bangladesh, the engagement of community-based organizations and local leaders has been shown to improve the performance and coverage of Community Health Workers (CHWs). Across these diverse settings, the evidence consistently demonstrates that the engagement of community structures, networks, and leadership plays a pivotal role in enabling community health workers to perform their jobs more effectively [88–90].

Trusting relationship as crucial enabler of HEWs and the HEP program is one of the important enablers identified in this review. According to the evidence, key qualities that HEWs should embrace in order to establish trust with the communities are effective Communication and Interaction skills, interpersonal collaboration abilities, strong work ethic, regular availability of HEWs at their work station, relevant work experience and perceived competency by the community was identified as strong enablers for their performance. When the community trusts HEWs, it encourages greater acceptance, engagement and participation in the program’s activities. This trust-based relationship facilitates open communication, allowing HEWs to better understand the community’s needs, concerns, and preferences. Moreover, the community is more inclined to offer practical and emotional support to HEWs they view as partners rather than outsiders, further enhancing the HEWs’ ability to perform their duties effectively. Beyond individual-level impacts, a trusting HEW-community relationship fosters a sense of ownership and commitment to the HEP at the community level. When the program is seen as a collaborative effort, rather than an external imposition, the community is more likely to take an active role in maintaining the program’s infrastructure, mobilizing resources, and advocating for its continued support. Evidence from elsewhere also supports that trusting relationships between community health workers and the communities they serve are crucial for the effectiveness of community health worker programs in low and middle-income countries (LMICs). In Bangladesh, the trust and rapport between Community Health Workers (CHWs) and the community members have been identified as key factors contributing to the performance and coverage of community health interventions [88–90]. A study in Uganda found that trust led to higher participation in health programs, with community members more likely to attend education sessions and engage in disease prevention activities [39]. Similarly, in Bangladesh, trust increased the acceptance and uptake of health interventions, such as vaccinations and maternal health services, resulting in improved health outcomes, including reduced infant mortality [91]. In Kenya, trusted CHWs communicated more effectively with community members, allowing for open discussions and tailored health advice [92]. In South Africa, CHWs with strong relationships saw higher adherence to health recommendations, such as medication compliance, due to trust in the guidance provided [93]. Furthermore, trust enhances access to vulnerable populations and reduces barriers within the health system. In India, trusted CHWs gained better access to groups like women and children, improving health equity [94]. In Nigeria, CHWs with trusting relationships acted as advocates, helping community members navigate the health system more effectively [89]. Additionally, in Peru, trust fostered a sense of empowerment and ownership over health initiatives, leading to sustainable improvements [95]. Thus, by building trust, community health workers can better understand the local context, tailor their interventions to community needs, and foster community engagement and ownership, ultimately leading to more effective and sustainable health service delivery.

The findings emphasize the crucial role of consistent availability of essential medical resources, equipment, and supporting infrastructure serves as a key facilitator, empowering HEWs to deliver timely and appropriate care to the communities they serve. Access to necessary tools such as drugs, contraceptives, vaccines, consumables, and vital medications like ORS, supplements, and anti-malaria medicines, along with basic equipment such as thermometers, fetoscopes, and stethoscopes, enables HEWs to meet community expectations and build trust. Additionally, properly furnished health post offices equipped with reliable electricity, telephone services, and water supply create a conducive environment for effective service delivery and serve as critical enablers. These facilities provide a conducive environment for HEWS to conduct health education sessions, consultations, and treatments, thereby enhancing the quality of care delivered. Reliable infrastructure, such as transportation and communication networks, is essential for the mobility and connectivity of health extension workers. Access transportation systems enable them to reach remote and underserved areas, while effective communication tools help them stay informed and connected with other healthcare providers, the community networks and their supervisors. Access to necessary stationery and a well-organized health post setup further facilitate HEW performance. Transportation and communication services, including coordinated ambulance services and the use of traditional ambulances when necessary, enable HEWs to respond swiftly to emergencies and provide comprehensive care. Thus, the governments need to ensure the supply chain of health posts and need to equip and support it well to enable the HEP to fulfill its mission of providing accessible and quality healthcare services to all Ethiopians. In several other settings, consistent availability of essential medical resources, equipment, and supporting infrastructure has been identified as a key facilitator that empowers community health workers to perform their duties effectively [39,96,97], with robust community health systems with adequate medical infrastructure, logistical support, and information systems have been linked to better outcomes for community health worker programs [39,96]. Frustrated community health workers due to stock outs or malfunctioning equipment can experience eroded trust in the communities they serve and undermine their effectiveness [97,98] and as a result, investing in ensuring consistent resource availability is often cited as a best practice for successful community health worker initiatives [94,99].

Improved living and work conditions have been shown to positively impact their performance and the overall effectiveness of the CHWs [40]. One study found that supportive work environments were critical for the motivation and performance of community health workers, including HEWs in Ethiopia [39]. Another study depicted that when HEWs work in supportive environments, they are more capable of delivering essential health services, resulting in better maternal and child health indicators [100]. Better living and working conditions contribute to higher retention rates among health workers. The retention of skilled workers is crucial for maintaining consistent and effective health service delivery [80]. The study found that providing better housing for health extension workers in Ethiopia led to a 15% increase in the number of households visited and a 20% improvement in the quality of health services delivered [54]. Another study indicated that health extension workers with higher job satisfaction due to improved working conditions and support had 25% lower attrition rates compared to their peers with poorer work environments [76].

The research highlighted the critical role that supportive living and working conditions play in enabling HEWs to fulfill their duties effectively. Several key insights emerge from the studies cited. First, supportive work environments, including adequate housing and other resources, are vital for motivating HEWs and enhancing their job performance. HEWs operating in such environments are better equipped to deliver essential health services, leading to tangible improvements in service delivery. Second, better living and working conditions contribute to higher retention rates among HEWs, and retaining skilled and experienced HEWs is crucial for maintaining consistent and effective HEP delivery within communities. Third, concrete interventions to improve HEW living conditions, such as providing better housing, can directly translate into measurable increases in household visits and the quality of services delivered, suggesting that relatively straightforward investments in HEW support can yield substantial dividends in community health outcomes. Finally, job satisfaction among HEWs, driven by improved working conditions and support, is associated with significantly lower attrition rates, highlighting the importance of holistically addressing the needs and concerns of this frontline health workforce to ensure their long-term commitment and performance. These findings emphasize the need for the Ethiopian government and program managers to prioritize the living and work environments of HEWs as a key strategy for strengthening the community-based HEP, as ensuring that HEWs have the resources, infrastructure, and support they need can unlock their full potential to deliver transformative primary healthcare services at the local level. There is substantial evidence that providing supportive living and working conditions for community health workers (CHWs) is crucial for enabling them to effectively carry out their duties and deliver high-quality services in low- and middle-income country (LMIC) settings [39,95,101]. These findings highlight the critical importance of investing in the work environments and personal circumstances of CHWs as a means of unlocking their full potential to serve their communities effectively. Policymakers and program managers in LMICs must prioritize ensuring that CHWs have the resources, infrastructure, and supportive supervision they need to carry out their roles successfully and contribute to improved population health outcomes.

The review identified a comprehensive enabling pathway framework that helps ensure Ethiopia’s HEP can deliver high-quality healthcare services across the country. This framework operates through multiple, interconnected mechanisms. First, it motivates and empowers the HEW workforce by improving their working conditions, living environments, and overall support systems, creating a more enabling atmosphere for them to carry out their duties effectively. Second, the framework leverages the power of community and stakeholder engagement, with coordinated involvement of these groups serving as a powerful enabler for the HEP. Third, it fosters trusted relationships and linkages among all actors in the system, particularly between HEWs and the communities they serve, which is crucial for program success. Fourth, the framework emphasizes capacity building and continuous system development, ensuring the HEP remains robust and responsive to evolving needs. Lastly, the availability of adequate resources and infrastructure is identified as a vital component, providing the functional capacity required to support high-quality service delivery. Together, these mutually reinforcing elements form a comprehensive enabling pathway that equips the HEP to extend primary healthcare access and improve population health outcomes across Ethiopia.

### Strengths and limitations

This review offers a comprehensive mapping of the local literature on HEWs and the Health HEP, from the program’s inception to the present. Covering a wide range of sources provides a thorough understanding of the enablers and facilitators identified across various types of evidence. A robust search strategy was employed, incorporating widely used databases to ensure broad coverage of the relevant literature.

However, inherent limitations of the scoping review methodology must be acknowledged, such as the lack of in-depth critical appraisal of individual studies. As a result, the quality and rigor of the included evidence were not systematically evaluated.

### Implications for Ethiopia and lessons from global community health programs

The scoping review underscores that optimizing Ethiopia’s HEP requires addressing multidimensional enablers, including enhancing HEWs motivation through career advancement and recognition, diversifying services to include context-specific and curative care, and strengthening community engagement through local structures like the WDA. Ethiopia should prioritize equitable resource distribution, supportive supervision, and improved living conditions for HEWs to sustain performance. Learning from global examples, Ethiopia could integrate gender-balanced community health workers to improve service accessibility, formalize roles of traditional birth attendants to leverage community trust, and adopt coordinated multi-stakeholder support systems to balance preventive and curative services. These strategies, aligned with the review’s findings, can accelerate progress toward UHC by ensuring HEP remains adaptive, community-driven, and resource-equipped to address Ethiopia’s diverse health needs.

## Conclusions

This scoping review synthesized key enabling and facilitating factors that enhance Health Extension Workers’ performance and Ethiopia’s HEP. Six major themes were identified: motivation factors, diversification and adaptation of HEP services and its modalities, continuous capacity building and ongoing support system, community and stakeholder engagement and leadership, trusting relationships, resource availability and conducive and supportive living and work environment. Intrinsic and extrinsic motivations are crucial for HEW performance, with job satisfaction, community connection, and career development opportunities being significant motivators. Diversifying and adapting HEP services and workforce, including integrating males and clinical nurses, can improve program reach and impact. Continuous capacity building and robust support systems, including collaborative supervision and mentorship, are essential for enabling HEW performance. Engaging communities through local structures, leadership, and stakeholder coordination enhances HEP outcomes. Trusting relationships between HEWs and the community foster collaboration and program success. Improved resources, facilities, and infrastructure at health posts, along with better living and working conditions for HEWs, significantly enable and enhance their performance and the program’s efficiency. Together, these elements form an enabling pathway to support Ethiopian HEP’s goal of expanding preventive and promotive services while integrating essential and basic curative elements in a balanced manner.

### Implications

#### Implications for policy.

Policymakers should recognize the critical role of intrinsic and extrinsic motivators in HEW performance and develop policies that support both aspects. This includes offering career development opportunities, competitive compensation, and recognition mechanisms. The diversification and adaptation of HEP to local contexts require decentralized planning and decision-making, empowering regional and district-level implementers. National guidelines and standards should be revisited to allow flexibility for local adaptations, enabling the program to respond to diverse socio-cultural and geographic settings. Integrating curative services should be carefully considered to maintain the program’s preventive and promotive focus. Policymakers should also strengthen the involvement of community volunteers, such as the Women Development Armies and traditional birth attendants, with clear roles and guidelines. The inclusion of males in the HEP also requires policy directions. Ensuring consistent availability of essential medical resources and supporting infrastructure at health posts is crucial for effective service delivery.

#### Implications for practice/Program.

For effective implementation, program managers should foster intrinsic motivation among HEWs by reinforcing their sense of societal obligation and belonging. This can be achieved by offering meaningful work, growth opportunities, and supporting community ties. Programs should incorporate intrinsic and extrinsic motivation strategies to sustain high performance. Diversifying the HEP workforce, including male HEP cadres, can strengthen the program and empower HEWs’ technical capacity. Continuous capacity building through tailored training, constructive feedback, and collaborative support is vital. Engaging community structures, networks, and leadership should be a core strategy, leveraging model families, local leaders, and community groups like the Women’s Development Army to enhance program effectiveness. Trust-building with communities requires HEWs to possess strong communication and interpersonal skills. Improving HEWs’ living and working conditions, including supportive housing and work environments, is necessary to enhance retention and performance. By implementing these strategies, the HEP can optimize HEW performance and improve healthcare service delivery in Ethiopia.

#### Implications for researches.

This review yields important findings with policy and practice implications. Yet, there are areas that require further studies to build knowledge bases as to what comprehensive enabling factors could exist to harness and foster HEP. Future research on Ethiopia’s HEP should focus on several key areas to enhance the program’s performance and sustainability. One critical area is the exploration of innovative community engagement strategies to increase participation and ownership, which can lead to improved health outcomes and program longevity. Additionally, assessing the long-term effects of capacity-building initiatives on the performance and retention of HEWs is important, emphasizing the significance of these initiatives in driving program success.

Research should also examine the interactions and collaborations between formal healthcare providers and various community volunteers, agents, and networks within the HEP ecosystem. Identifying strategies to enhance collaboration, synergy, coordination, and leadership commitment is essential for optimizing program outcomes. Furthermore, investigating gender dynamics within the HEP can provide valuable insights into addressing gender disparities and ensuring equitable access to healthcare services for all community members. The conceptualized enabling pathways framework of the HEP should be further explored through rigorous statistical research to model and assess the impact of these enabling elements on HEW/HEP performance. Future analyses should also synthesize a comprehensive range of barriers and challenges that affect the implementation of the HEP. Such research can help identify the most effective strategies and interventions to support and strengthen the HEP, ultimately contributing to improved health outcomes and increased access to healthcare across Ethiopia.

## Supporting information

S1 File Search Stratagy_PubMed.docx. (DOCX)

S2 File PRISMA_2020_checklist_PRISMA-ScR CHECKLIST.docx. (DOCX)

S3 File Data set (grand summary). (XLSX)

S4 File PRISMA_2020_checklist Abstract. (DOCX)
