# Supplementary material for: Multidimensional performance enablers of Ethiopian community-based health extension program: A scoping review
Source: PLoS One. 2025 Jun 5;20(6):e0324377. doi: 10.1371/journal.pone.0324377 (PMC12140219; doi:10.1371/journal.pone.0324377)
Supplement: S1 File — (DOCX) [file pone.0324377.s001.docx]

Supplement I: Search Strategy (PubMed)

| Search | Query | Hits |
| --- | --- | --- |
| 1 | **All fields:** Health extension worker OR Health extension workers OR HEWs OR Primary healthcare workers OR Primary health care workers OR "Community Health Workers"[Mesh] OR Health extension Program OR HEP OR Community Health Program OR Community Health Worker OR CHWs OR Community Health Agent OR Lay Health Worker* OR Community Health Educator OR Community health promoter OR Volunt* Health Workers OR frontline health workers OR "Community Health Workers"[Mesh] | 2,634,596 |
| 2 | **All fields:** Performance OR Work Performance OR Performance evaluation OR Performance indicator* OR Performance measure* OR Evaluation OR measure* OR indicator* OR effective* OR Efficiency OR Impact OR "Program Evaluation"[Mesh] OR Efficien* OR Key Performance Indicator OR KPI OR (Productivity) AND (Performance) OR Work Performance OR Performance evaluation OR Performance indicator* OR Performance measure* OR Evaluation OR measure* OR indicator* OR effective* OR Efficiency OR Impact OR "Program Evaluation"[Mesh] OR Efficien* OR Key Performance Indicator OR KPI OR Productivity | 14,603,558 |
| 3 | **All fields:** Ethiopia OR "Ethiopia"[Mesh] OR Tigray OR Afar OR Amhara OR Oromi* OR Somali OR Benshangul OR Benishangul OR South nation nationalities and peoples Region OR SNNPR OR Gambella OR Gambela OR Harari OR Diredawa OR Dire-Dawa OR Dire Dawa OR Addis Ababa OR Sidama | 39,528 |
| 3 | 1 AND 2 AND 3 | 7,491 |
| 4 | Filters applied: Humans, English, | 5,439 |
| Search date: 01/01/2024 | | |
